# The Role of Artificial Intelligence in the Detection and Implementation of Biomarkers for Hepatocellular Carcinoma: Outlook and Opportunities

**DOI:** 10.3390/cancers15112928

**Published:** 2023-05-26

**Authors:** Arian Mansur, Andrea Vrionis, Jonathan P. Charles, Kayesha Hancel, John C. Panagides, Farzad Moloudi, Shams Iqbal, Dania Daye

**Affiliations:** 1 Harvard Medical School, Boston, MA 02115, USA; arianmansur@hms.harvard.edu (A.M.); john_panagides@hms.harvard.edu (J.C.P.); 2 Morsani College of Medicine, University of South Florida Health, Tampa, FL 33602, USA; vrionis@usf.edu (A.V.); charles1@usf.edu (J.P.C.); 3 Department of Radiology, Massachusetts General Hospital, Boston, MA 02114, USA; khancel@mgh.harvard.edu (K.H.); fmoloudi@mgh.harvard.edu (F.M.); siiqbal@mgh.harvard.edu (S.I.)

**Keywords:** artificial intelligence, machine learning, deep learning, biomarkers, liver cancer, hepatocellular carcinoma

## Abstract

**Simple Summary:**

Liver cancer is a major health problem worldwide, and its early detection and management are imperative for improving patient outcomes. Biomarkers have great potential to aid in the early detection and management of liver cancer. However, biomarker detection and implementation in management can be quite challenging. Artificial intelligence has shown promise in both the detection and use of biomarkers in liver cancer management, making it a valuable tool for research and future clinical implementation. The purpose of this review is to provide an overview of the current state of AI-based biomarker research in liver cancer. The findings from this research highlight the important role that AI could play in improving the accuracy and reliability of biomarker detection and its implementation for liver cancers.

**Abstract:**

Liver cancer is a leading cause of cancer-related death worldwide, and its early detection and treatment are crucial for improving morbidity and mortality. Biomarkers have the potential to facilitate the early diagnosis and management of liver cancer, but identifying and implementing effective biomarkers remains a major challenge. In recent years, artificial intelligence has emerged as a promising tool in the cancer sphere, and recent literature suggests that it is very promising in facilitating biomarker use in liver cancer. This review provides an overview of the status of AI-based biomarker research in liver cancer, with a focus on the detection and implementation of biomarkers for risk prediction, diagnosis, staging, prognostication, prediction of treatment response, and recurrence of liver cancers.

## 1. Introduction

Given the lack of randomized clinical trials evaluating the efficacy of screening for primary hepatocellular carcinoma (HCC), current practices rely on retrospective observational data and in silico models to inform clinical guidelines [1]. The current clinical surveillance strategy relies on the identification of high-risk individuals who would likely benefit from screening, which specifically includes (1) patients with cirrhosis and Child–Pugh class A or B, (2) patients with cirrhosis and Child–Pugh class C only if awaiting liver transplantation, (3) patients without cirrhosis who have hepatitis B virus (HBV) infection and have active hepatitis, a family history of HCC, and are African or African American, a male Asian over 40 years of age, a female Asian over 50 years of age, and sometimes (4) patients with chronic hepatitis C virus (HCV) who have advanced liver fibrosis (stage F3) [2,3,4,5,6]. The accepted screening regimen including semiannual hepatic ultrasonography with or without serum alpha-fetoprotein (AFP) measurement, a widely recognized tumor marker, has set a precedent for the identification of HCC biomarkers in both imaging and laboratory data, despite a lack of consensus for the appropriateness of contemporaneous AFP measurement and the applicability of screening in patients without cirrhosis [6,7]. Studies have shown variable sensitivity and specificity for AFP in diagnosing HCC, with specificity as high as 99% for AFP greater than 400 ng/mL, but with low sensitivity of 32% and lower thresholds of 20–100 ng/mL, with sensitivity of 61% and specificity of 86% [8]. Multiple categories of HCC biomarkers have been proposed, including imaging features (e.g., quantitative and qualitative features such as tumor size, volume, morphology, enhancement patterns, and vascular invasion data from MRI or CT), quantification of endogeneous serum proteins and/or nucleic acids, circulating tumor-derived molecules, and genomic expression data, all of which may be combined with clinical parameters to generate a multicompartmental biomarker panel [9,10]. However, there is no consensus regarding if and how these biomarkers should be combined in a manner that is clinically useful or regarding the establishment of a pipeline to discover new biomarkers.

Artificial intelligence (AI) has emerged as a computational technique to identify and evaluate candidate biomarkers for HCC [11,12,13,14]. In general, AI approaches relevant to biomarker detection can be divided into computational search algorithms, machine learning (ML), and deep learning (DL; e.g., convolutional neural networks, CNNs) [15]. Computational search algorithms utilize structured, iterative approaches to evaluate a list of variables, while ML techniques incorporate an inherent feedback mechanism to adjust model parameters during a training period (with subsequent validation during a testing period). These approaches often implement supervised learning, a technique that relies on labeled variables and outcome data (i.e., given a target outcome of diagnosing HCC on an imaging study, the label would establish the ground truth of the presence or absence of HCC from the radiology report). Random forest analysis, which is a type of supervised ML algorithm, can be used to create an ensemble of decision trees that collectively make predictions, with each decision tree being trained on a random subset of the data. In contrast, deep learning and associated CNNs often implement unsupervised learning, a technique that accepts unlabeled data and classifies cases based on shared features [15,16]. Because unsupervised learning often identifies features that may not be discernable to the human observer, CNNs often require additional interpretation, but may generate unique insights, including variables previously not emphasized in explicit models [16,17]. Additional interpretations often involve identifying specific features or patterns that can contribute to the model’s predictions and can be achieved with techniques such as feature visualization and gradient-based attribution methods. These features help provide a better picture of the numerous clinicopathological aspects of the disease and help inform predictions.

AI was first used to identify imaging features that would augment the HCC response prediction to minimally invasive therapies (e.g., transarterial chemoembolization, TACE) in 2019 [18]. This work has been expanded to evaluate broad data categories including laboratory values, clinical variables, and imaging features as covariates in TACE-response prediction models in order to better utilize biomarkers for the detection of HCC, prediction of treatment, surveillance, and prognostication [9,19]. This review focuses on the role of artificial intelligence in detecting and utilizing biomarkers in four HCC domains: detection, prediction, surveillance, and prognostication.

## 2. AI-Assisted Biomarker Detection

While multiple biomarkers are known for diagnosing liver cancers, advances in technology, especially with the generation of large biological multi-omics datasets and AI algorithms, have expanded the potential for biomarker detection [15,20,21]. Here, we highlight the discovery process of novel biomarkers through AI techniques before diving into the implementation and evaluation of their clinical utility.

Kaur et al. used ML and large-scale transcriptomic profiling data from 2316 HCC and 1665 nontumorous tissue samples to identify three platform-independent diagnostic genes—*FCN3* (downregulated in HCC), *CLEC1B* (downregulated in HCC), *PRC1* (upregulated in HCC)—that were able to detect HCC in both training and validation datasets with good accuracy (93–98%) and show prognostic potential [22]. Gui et al. similarly used ML to identify genes, both known HCC-relevant ones (e.g., MT1X, BM1, and CAP2) as well as those not previously known to be closely related (e.g., TACSTD2), that could separate tumor and nontumorous samples [23]. Differences in panels amongst studies are likely due to heterogeneous datasets and heterogeneous genetic profiles in individual tumor samples.

Multiple studies have analyzed RNA-seq data through machine learning to identify novel transcript biomarkers. Applying this technique, Gupta et al. discovered three new biomarkers—PARP2–202, SPON2–203, and CYREN-211—differentiating normal hepatic tissue from HCC tumor tissue [24]. The first two were noted to be protein-coding, while the last one was noncoding, illustrating that biomarker value does not always depend on the coding status of a transcript and these noncoding transcripts may still have crucial regulatory roles. Additionally, they found that the known biomarkers for HCC that they had gathered from an exhaustive literature review (Gupta et al. Table 1) held the greatest predictive value, but the addition of the above transcripts contributed to improved overall accuracy. Gholizadeh et al. also studied mRNAs via machine learning to develop a screening program differentiating normal tissue from HCC tissue, resulting in three diagnostic biomarkers (CYP2E1, ARK1C3, AFP) and four additional prognostic markers (SOCS2, MAGEA6, RDH16, RTN3) [25]. Zhang and Liu carried out an analysis utilizing high-throughput omics data provided by the Cancer Genome Atlas (TCGA) [26]. They focused on the feature selection process of machine learning to select six key feature subsets to serve as robust biomarkers. The authors looked at the effectiveness of identified biomarkers, with some important genes that they found to be overlapping, including SKAP1, EPHB1, STC2, CDHR2, FAM134B, MUC6, PHOSPHO1, and OXT, each associated in some way with the development of HCC. Providing evidence for this technique, these biomarkers have been shown to closely relate to HCC establishment and progression. Zhao et al. similarly implemented machine learning, specifically random forest algorithm, to uncover differentially expressed miRNA and mRNA biomarkers for HCC diagnosis [27]. Five miRNAs (hsa-miR-10b-5p, hsa-miR-10b-3p, hsa-miR-224-5p, hsa-miR-183-5p and hsa-miR-182-5p) along with the respective mRNAs they target (SFRP1, EDNRB, NR4A3, FHL2, NKX3-1, IL6ST and FOXO1) were discovered and linked to HCC tumorigenesis, providing both diagnostic and prognostic information. These studies are summarized in Table 1. Moving beyond the evaluation of normal tissue versus HCC tissue, one meta-analysis of gene expression profiles identified biomarkers to differentiate between HCC and cholangiocarcinoma via transcriptome networks [28].

Outside of RNA sequencing, many other methods of biomarker detection through AI machine learning have been successfully employed. Circulating cell-free DNA (cfDNA) has emerged as a promising biomarker for early HCC and monitoring disease progression. Machine learning analysis of the genetic changes in circulating tumor DNA (ctDNA), a component of cfDNA, break down tumor heterogeneity into identifiable molecular processes (e.g., mutations, epigenetic changes) that can be targeted through precision oncology, given that precision oncology uses biomarkers in decision making for treatment and predicting prognosis [7]. Furthermore, epigenetic changes, such as DNA methylation at 5-methycytosine sites, occur in early tumorigenesis and thus can aid in early detection of cancer. Methylation is tissue-specific, which can enable this biomarker to detect the site of a cancer through liquid biopsy. Lee et al. integrated cfDNA expression profiles through a machine learning algorithm in the establishment of a new scoring system that accurately predicted clinicopathological characteristics of patients with HCC [29]. CfDNA was used to predict pathological features of HCC, including stage, lymphovascular invasion (LVI), size, and multifocality. High cfDNA in the plasma was found to be associated with LVI and predicted the number of tumors present. The plasma concentration of cfDNA was strongly correlated with the size of tumors.

Cis-diol metabolites are vital components of physiological processes involving energy storage and signal transduction. Their dysregulation has been noted in many complex diseases, such as cancer and diabetes. A 2023 study demonstrated the ability of machine learning to interpret cis-diol metabolic fingerprinting for precise diagnosis of primary liver cancer [30]. Ge et al. recently discovered VIPR1 as an early diagnostic biomarker through machine learning from microarray datasets [31]. This gene is involved in glycogen metabolism and immune system regulation. Poon et al. developed classification trees and neural networks to detect serum biomarkers and clinical factors to diagnose HCC and differentiate from liver cirrhosis [32]. Their model showed that AFP in combination with their neural network model improved the diagnostic sensitivity for HCC from 60% to 73.8% (*p* < 0.05). Additionally, their neural networks were able to detect HCC in patients without much elevation in serum AFP levels (below 500 ng/mL) [32].

ML in metabolomics approaches (e.g., liquid chromatography–mass spectrometry (LC/MS)) has also allowed for the identification of metabolic biomarkers. Liu et al. utilized nuclear magnetic resonance (NMR), LC-MS, and random forest analysis to analyze the serum metabolic profiles of patients with HCC/cirrhosis and healthy volunteers [33]. The study found 32 biomarkers, including perturbations in amino acid catabolism, bile acid metabolism, citrate cycle, fatty acid oxidation, ketone body synthesis, phospholipid metabolism, and sphingolipid metabolism, associated with HCC with 100% sensitivity in detecting patients with HCC (100% specificity in first validation set and 94.7% in the second), even when AFP levels were less than 20 ng/mL. Similarly, Lin et al. used LC-MS, random forest analysis, and logistic regression analysis to identify 15 metabolites that could distinguish patients with HCC and negative AFP from cirrhosis and healthy volunteers [34]. They developed a three-marker model of metabolites to identify AFP-negative HCC. The model showed sensitivity of 72.7%, specificity of 92%, and AUC of 0.91. Liang et al. applied LC-MS and ML to urine samples in patients with HCC to discover 15 differential metabolites, five of which were able to diagnose HCC with a sensitivity of 96.5%, specificity of 83%, and AUC of 0.90 [35].

AI has also paved the way for data mining in the literature to detect biomarkers. Chang et al. conducted biomedical text mining with a CNN to extract biomarkers from the literature [14]. The researchers created MarkerHub, which contains 2128 candidate biomarkers from 2008 to 2017.

## 3. The Role of AI in Facilitating Biomarker Implementation in Liver Cancers to Predict the Risk of Liver Cancers

Most HCC cases develop in the setting of underlying cirrhotic hepatic disease. Therefore, the risk and/or presence of risk factors for cirrhosis may inform the risk of future HCC development. Relevant conditions include nonalcoholic fatty liver disease (NAFLD), nonalcoholic steatohepatitis (NASH), hepatitis B virus (HBV) infection, and hepatitis C virus (HCV) infection [16].

AI approaches have been proposed to increase the accuracy of predicting HCC risk and are summarized in Table 2. Liang et al. developed a DL tool that incorporates data from electronic health records, imaging, histopathology, and molecular biomarkers into a CNN to predict the risk of being diagnosed with HCC in 1 year [36]. This study included 47,945 individuals, of whom 9553 had HCC, and the AUC for predicting risk of HCC 1 year in advance was 0.94 (95% CI 0.937–0.943). Singal et al. used regression analysis as well as ML algorithms to design models to predict the development of HCC in 422 patients with Child A or B cirrhosis that were independently validated in the cohort from the Hepatitis C Antiviral Long-Term Treatment against Cirrhosis (HALT-C) trial [37]. The ML algorithm was found to have higher accuracy in identifying patients at high risk of developing HCC when compared to regression analysis and the previously published HALT-C model. These algorithms incorporated demographic data, clinical data, and laboratory values (AST levels, ALT levels, presence of ascites, bilirubin, baseline AFP, and albumin) to identify the patients who developed HCC. Konerman et al. examined longitudinal data in the HALT-C trial as a predictor of clinical outcomes, including HCC, in the following year [38]. The predictive models were built using logistic regression and ML (specifically random forest and boosting) and were applied to predictor variables, including AST, ALT, AST/ALT, total bilirubin, albumin, alkaline phosphatase, APRI, AFP, and creatinine. The longitudinal models had significantly higher performance than the baseline models, and the longitudinal ML models had negative predictive value of 94% for fibrosis progression and liver-related clinical outcomes, including HCC.

Ioannou et al. evaluated a DL recurrent neural network (RNN) in 48,151 patients with hepatitis C virus-related cirrhosis in the national Veterans Health Administration and found that their DL RNN models had significantly greater performance than linear regression models in addition to detecting patients at high risk of progressing to HCC [12]. Dawuti et al. used surface-enhanced Raman spectroscopy (SERS) and a support vector machine (SVM) algorithm to screen patients with liver cirrhosis and HCC via biomolecules in urine and the metabolism of specific nucleic acids and amino acids [39]. The study found that the combination of the urine SERS spectra with the SVM algorithm could detect liver cirrhosis and HCC (with a higher diagnostic sensitivity than serum AFP).

## 4. The Role of AI in Facilitating Biomarkers to Diagnose Liver Cancers

**Figure 1:**
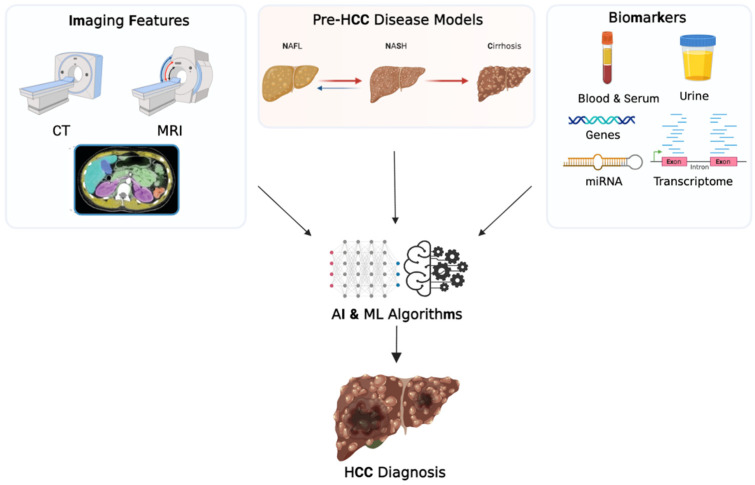
Schematic representation of the role of artificial intelligence in the diagnosis of hepatocellular carcinoma. Abbreviations: CT, computed tomography; MRI, magnetic resonance imaging; NAFL, nonalcoholic fatty liver disease; NASH, nonalcoholic steatohepatitis; miRNA, microribonucleic acid; AI, artificial intelligence; ML, machine learning. Created with BioRender.com, accessed on 13 March 2023.

Traditional approaches for the diagnosis of HCC may include clinical, radiological, and laboratory analysis, and AFP is the standard biomarker for diagnosing HCC. In addition to predicting risk, AI has the potential to improve the traditional diagnosis methods by combining multiple features. Figure 1 shows an example of the various inputs that AI and ML algorithms implement for HCC diagnosis.

An early assessment of neural network utility to distinguish HCC in patients with underlying liver cirrhosis was assessed by Poon et al. in 2001 [32]. Proposed biomarkers included serological values (i.e., AFP, alpha-1 antitrypsin (A1AT), alpha-2 macroglobulin (A2MG), thyroxine-binding globulin (TBG), transferrin, and albumin) in addition to clinical parameters such as sex and age. Compared to AFP alone, the utilization of neural networks improved the diagnostic sensitivity from 60% to 73.8% (*p* < 0.05) with specificity of 88.2%. Sato et al. also combined multiple serum biomarkers that are clinically available and used a graphical user interface ML-based predictive model to diagnose HCC [40]. The results showed an AUC of 0.94 and a more accurate classification rate for combined biomarkers when compared to using a single tumor marker for prediction. Another study used a combination of serum protein fingerprinting (using mass spectrometry) and artificial neural network models in the diagnosis of HCC. Wang et al. trained an artificial neural network with data from 70 participants and tested the effectiveness in predicting HCC [41]. The model had 100% sensitivity and specificity in comparing patients with HCC against patients without. Comparing HCC and liver cirrhosis had 88.2% sensitivity and 100% specificity. This shows promise for the use of AI, particularly using neural networks, in the accurate diagnosis of HCC at an early stage. Ksiazek et al. used 10 different machine learning algorithms and compared their accuracy with and without feature selection to detect HCC from a clinical dataset [42]. The ML algorithms were support vector classifier (SVC), NuSVM, LinSVM, Relog, Multilayer Perceptor 1 (MLP1), linear discriminant analysis (LDA), random forest (RF), k-nearest neighbor (KNN), quadratic discriminant analysis (QDA), and GaussNB. Quantitative features in the dataset included some relevant biomarkers such as AFP, AST, gamma-glutamyl transferase, and alkaline phosphatase. Of the ML techniques, SVM (SVC) with feature selection resulted in the most accurate detection of HCC for early diagnosis with an accuracy of 0.88. These findings suggest that future studies should employ a wide range of features, including clinical, pathological, and imaging, and have several AI techniques to choose from.

## 5. The Role of AI in Facilitating Biomarkers to Stage Liver Cancers

Consistent staging of liver cancer has proved an elusive and challenging task due to the vast molecular heterogeneity among hepatic tumors. The TNM staging system, based on tumor size, lymph node spread, and presence of metastasis, is the most widely adopted cancer staging system. The Barcelona Clinic Liver Cancer (BCLC) staging system is thought to outperform the TNM system in determining patient prognosis, through inclusion of liver function and performance status in the assessment. However, neither system is universally in place at this time.

Although the BCLC staging system is instrumental in guiding recommendations for guiding therapies, unfavorable outcomes remain higher than hoped [43]. In response to this problem, Bagante et al. proposed a strategy for advancing staging system accuracy, which in theory would subsequently improve patient prognosis, treatment, and overall outcomes [44]. They advocated for the integration of molecular classifications and biomarkers with clinical data for staging purposes and illustrated the improved prognostic accuracy in comparison with standard staging systems. In a similar effort, Xu et al. looked to identify biomarkers specific for BCLC staging [45]. This study recognized the heterogeneity of tumor molecular genetics within a single clinical stage and aimed to elucidate further classifications within stages to identify optimal candidates for liver resection. The identification of biomarkers associated with higher BCLC staging and worse overall survival (OS) in patients with HCC was hypothesized to better reveal optimal surgical candidates with decreased probability of recurrence. The results of this investigation included 13 specific hub genes (*TIGD5*, *C8ORF33*, *NUDCD1*, *INTS8*, *ZNF623*, *STIP1*, *HSP90AB1*, *DSCC1*, *POP1*, *ARHGAP39*, *PRKDC*, *YDJC*, and *PUSL1*) that were closely linked to tumor stage and survival time, as demonstrated in Table 3. Patients with overexpression of these genes were hypothesized to exhibit worse prognosis after surgery and should potentially be advised away from treatments such as curative surgery. Although further studies are needed to validate this theory, the integration of biomarkers in the staging of liver cancers provides an exciting new approach for researchers.

Kaur et al. utilized machine learning algorithms via transcriptomics to separate liver cancer into early and late stages [46]. In sum, 21 CpG methylation sites were implicated in differentiating early stage from late stage with an AUC of 0.78 on validation data, and 30 identified RNA transcripts were found to be upregulated in late stages, with an AUC of 0.77 on the validation dataset. These upregulated genes are notably involved in cell division processes and transcriptional regulation, contributing to chromatid stabilization, phosphorylation of deoxyribonucleosides, and elongation of precursor proteins, among other roles. Utilizing hybrid prediction models combining the 51 total features of CpG methylation sites and RNA transcripts, classifying samples into early and late stages was performed with 78% accuracy and an AUC of 0.82 on the independent dataset. Estevez et al. utilized random forest ML to demonstrate that cytokines may be used to stratify HCC according to the underlying hepatitis serovariant (if present) versus nonviral etiologies. Future studies will need to consolidate this information and provide validation of biomarker integration with tumor staging systems.

## 6. The Role of AI in Facilitating Biomarkers to Prognosticate Liver Cancers

The ability to determine the time course of a patient’s pathology is key in deciding which therapies to introduce and for the overall planning of the patient’s long-term treatment plan. As AI and ML models continue to improve and gain traction in the medical sphere, research on their application and efficacy for cancer treatment will be paramount. Lai et al. performed a systematic review to assess the role of AI in predicting survival and prognosis following treatment of HCC [47]. In all of the nine studies, AI demonstrated superior predictive prognostic performance compared with the traditional linear systems of analysis.

The search for tissue and serum biomarkers that can provide prognostic value in liver cancer can be facilitated through the use of ML computation. Liang et al. introduced in a preprint manuscript an interpretable human-centric deep learning software that assists pathologists in discovering new biomarkers from validated deep learning models [48]. Through the use of whole-slide tissue images from patients in the Cancer Genome Atlas Liver Hepatocellular Carcinoma and the Beijing Tsinghua Changgung Hospital datasets and delineated tissue spatial distribution characteristics from them, their program was able to localize, characterize, and verify potential biomarkers. Features from the whole-slide scans of proposed biomarkers were verified via a multiclass tissue segmentation network, PaSegNet, which obtained multiclass tissue probability heatmaps. From this, for high-precision prognosis, they built a system—MacroNet—composed of a convolution feature extract and a multilayer perceptron with a batch-normalize layer. Ultimately, this team was able to discover that levels of tumor necrosis in liver cancer have a strong relationship with patient prognosis. From this discovery, they formulated two clinically independent indicators to proxy this level: tumor necrosis distribution and the necrosis area fraction. Brar et al. suggested the use of a detailed systemic approach incorporating ML to survey several databases for possible biomarkers with prognostic potential in liver cancer [49]. They included peer-reviewed literature that had applied ML to molecular tissue data from liver cancers to predict prognosis for patients with primary HCC, which varies from the usual methodology of radiography to diagnose and prognosticate liver cancer. They found several different ML techniques that were incorporated, including DL, unsupervised learning, and supervised learning, across many arenas, such as proteomics, epigenomics, and metabolomics. Their results found no common genes amongst the prognostic signatures initially identified, but this work lays the groundwork for similar studies to explore the impact of machine learning in liver cancer prognosis as the body of current evidence continues to grow.

The use of AI and ML algorithms in the prognostication of liver cancer has been exceedingly common as the technology becomes more refined and efficient. Chaudhary et al. employed a deep learning-based model that utilized RNA sequencing, miRNA sequencing, and methylation data from HCC tissue samples in order to predict HCC survival prognosis [50]. The software was able to differentiate the patients into two subgroups and show significant survival differences (*p* < 0.001) between the two. They went further and associated the aggressive subgroup with TP53 inactivation mutations, activated Wnt and Akt signaling pathways, and higher expressions of biomarkers KRT19 and EPCAM, and validated their model with several external datasets. This study was one of the first to employ a multi-omics AI model to identify HCC biomarkers that were significantly associated with patient survival and prognosis. Tsilimigras et al. employed a similar strategy, where patients who were post-hepatectomy for BCLC-0, A, and B HCC were sorted into groups relative to their overall survival through a machine learning classification and regression tree model [51]. The model selected for both preoperative and postoperative markers that could be used to predict patient prognosis. It found that AFP and a Charlson comorbidity score were the most predictive preoperative factors for BCLC-0 and A patients, and radiologic tumor burden score proved to be the most predictive for BCLC-B patients. Postoperatively, the model found that the radiologic tumor burden score was still the best for BCLC-B patients, but found lymphovascular invasion the best for BCLC-0 and A. Cumulatively, they found the tumor burden score was the best at independently predicting overall survival among BCLC-0/A (HR: 1.04, 95% CI: 1.02–1.07) and BCLC-B (HR: 1.13, 95% CI: 1.06–1.19) patients undergoing hepatectomy. Huang et al. investigated the similar problem of predicting HCC survival with AI, but decided to employ a bidirectional deep neural network to integrate both patient DNA and RNA data [52]. Through the use of optimal clusters as labels, they developed a support vector machine model to predict survival. The model learned DNA methylation and RNA-sequence data in patients with HCC and categorized the patients into poor and good prognosis subgroups. This subgroup classification was found to be an independent prognostic factor, and that the patients who were sorted into the good-prognosis subgroup were actually found to live longer than the poor-prognosis subgroup. The use of bidirectional deep neural networks can be a novel tool in the armamentarium of clinicians determining the optimal therapy for patients with HCC. Another method devised to predict HCC prognosis is the creation of a prognosis score for patients. Tohme et al. decided to do this through the use of artificial neural networks and classification tree analysis to create a signature score based on the genomic profiles of patients with HCC [53]. They performed the neural network analysis on 75 of the most significant genes predicting disease-free survival as obtained from the Cancer Genome Atlas and used the tree analysis for producing the score and a Cox regression analysis to determine the actual prognostic value of the scoring system. They identified three genes, NRM, STAG3, and SNHG20, that were found to provide adequate prognostic value and used them to create the scoring system. Based on various expressions of the genes, the model was able to find a pattern in the disease-free survival as well as overall survival of the patients, with higher scores being associated with significantly shorter survival (*p* < 0.01). The literature discussed above shows that several novel studies have been published demonstrating the current capabilities of artificial intelligence in providing accurate and reliable prognostication for liver cancers, namely, HCC.

## 7. The Role of AI in Facilitating Biomarkers to Predict the Efficacy and Response to Treatment

Biomarkers play an imperative role in predicting the efficacy and response to treatment in liver cancers. AI is starting to facilitate this role in recent years (Table 4). For instance, Hsu et al. utilized a decision tree-based survival predictive model using a random forest algorithm to assess how serum biomarkers, such as AFP, albumin–bilirubin (ALBI) grade, and circulating angiogenic factors predict the efficacy of lenvatinib in patients with unresectable HCC [54]. The study found that a reduction in AFP ≥ 40% from baseline within 8 weeks posttreatment was associated with a higher objective response rate (ORR). Results from their decision tree-based models led to the identification of patients with high, intermediate, and low ORRs (*p* < 0.001), in addition to determining that baseline AFP was the most critical variable for predicting overall survival, with ALBI grade second and fibroblast growth factor-19 (FGF19) third. Similarly, Ma et al. used five ML algorithms to predict the combination treatment response to TACE and lenvatinib in patients with unresectable HCC using demographic features, pretreatment serum biomarkers, and tumor features, and their predictive models had AUCs between 0.74 and 0.91 [55].

Zhong et al. constructed a nomogram and artificial neural network (ANN) to compare the prognostic performance of ALBI grade and Child–Turcotte–Pugh (CTP) score in 838 patients (*n* = 548 in the training cohort; *n* = 115 in the first validation cohort; *n* = 175 in the second validation cohort) with HCC who underwent TACE [56]. Their study identified ALBI grade and CTP score as significant risk factors associated with OS in the training cohort, and these had similar prognostic performance in external validation cohorts when determined based on nomograms. Their ANN found that ABLI grade had greater significance than CTP score in predicting survival, suggesting that ALBI grade may eventually be a viable alternative, though more research is needed [58]. In another study, Morshid et al. analyzed the use of a fully automated ML algorithm that incorporated pretreatment quantitative CT image features and clinical information to predict the response of TACE in 105 patients with HCC [18]. Results from their model that combined BCLC stage plus quantitative image features showed a predication accuracy of 74.2% (95% CI: 64–82%) while the model with BCLC stage alone had a prediction accuracy of 62.9% (95% CI: 52–72%). Abajian et al. used ML, magnetic resonance (MR) imaging, and clinical data to predict the outcomes of 36 patients with HCC who were treated with TACE [19]. The study found that both the LR and random forest models could predict treatment response with an overall accuracy rate of 78%. Peng et al. used DL with a residual CNN (ResNet50) on CT imaging data to predict the treatment response of 789 patients with intermediate-stage HCC who underwent TACE with good accuracy [57]. Further work on the trends of AI, radiomics, and locoregional therapy in real of HCC treatment response are summarized in an article written by members of the LI-RADS Treatment Response (TR LI-RADS) work group and associates [59].

## 8. The Role of AI in Facilitating Biomarkers to Predict the Recurrence of Liver Cancers

The ability to predict which patients are at an increased risk of tumor recurrence is also invaluable information to have when conducting long-term treatment plans. These data can help to tailor patient’s therapies more effectively and can ideally provide clinicians and patients with a more detailed picture of the disease course. AI can tremendously impact patient outcomes and their treatment plans through more accurate predictions of tumor recurrence. Rodriguez-Luna et al. utilized an ANN in combination with genotyping for microsatellite mutations or deletions to predict HCC tumor recurrence in 19 patients who underwent liver transplantation with a discriminatory power of 89.5% [60]. Shen et al. built and subsequently verified an HCC tumor recurrence prediction model using public databases and ML to formulate genomic signatures that may be associated with recurrence [61]. Through a combination of AI and traditional statistical methods such as chi-squared tests and survival analysis, their model was able to achieve an accuracy of tumor recurrence prediction of 74.2%. Similarly, Fu et al. built an AI model that combined machine learning-derived lncRNA signatures with TNM stages and AFP values to predict early HCC recurrence [62]. They employed three widely used machine learning methods, LASSO, random forest and SVM-RFE, as well as multivariate Cox analysis to select the correct lncRNA signatures. This multivariate analysis suggested that the machine learning risk score (HR = 1.5, *p* = 0.015), AFP values (HR = 1.74, *p* = 0.012) and TNM staging (HR = 2.01, *p* = 0.01 for stage III + IV) were all independent recurrence indicators of HCC. Similar studies have been carried out where multiple gene signatures were created and validated with AI assistance and machine learning algorithms in the hopes of prediction of HCC recurrence [63,64,65].

Another biomarker that has been studied with respect to liver cancer recurrence is cfDNA. Several studies have confirmed that serum levels of cfDNA are highly associated with the potential for tumor recurrence [66,67]. However, the clinical implication of this biomarker has been limited due to the large volume of blood required for detection and the difficulties associated with processing [68]. Lee et al. made an attempt to detect and capture cfDNA using small amounts of blood and to investigate the prognostic potential of the cfDNA for HCC [29]. They employed a ML technique that has been previously described in a bead-based liquid biopsy assay to collect and analyze their cfDNA sample, and to create a cfDNA predictive score. They found that a higher score was associated with a poorer prognosis in recurrence-free survival in patients treated with transarterial chemoembolization (TACE). The mean recurrence-free survival was significantly higher in the cohort of patients who had a cfDNA score lower than the median score than the cohort with a score higher than the median score (26.5 ± 5.2 versus 15.9 ± 4.0 months, *p* = 0.061). Further, the score was able to predict the occurrence of marginal and multifocal HCC recurrences. The patients with scores lower than the median had multifocal recurrence-free survival times of 54.0 ± 4.6 versus 23.2 ± 5.5 months (*p* = 0.001) in the higher-than-median cohort. The same trends were seen in the marginal recurrence-free survival times, with the lower-than-median cohort having survival times of 41.1 ± 6.3 versus 27.4 ± 6.3 months (*p* = 0.061) in the higher-than-median cohort. Their cfDNA score was able to predict multifocal and marginal recurrence more reliably than other biomarkers used in the study, including AFP, albumin, and total protein (HR = 1.728, *p* = 0.005).

The literature presented above is to illustrate that AI and ML software can be effectively utilized in predicting a patient’s risk of liver cancer recurrence with accuracy and reliability. As sample sizes begin to grow, the models will be tested to determine if they are able to accommodate the increase in data input and will continue to be refined into the future as newer technologies and methods continue to be researched.

Some studies have proposed that circulating tumor cells in peripheral blood can be a promising candidate for predicting tumor recurrence, given that they may reflect the overall aggressiveness of the cancer [69]. Sun et al. studied circulating tumor cells that were EpCAM-positive in patient’s peripheral blood to determine if they can reliably predict HCC tumor recurrence [70]. They tested the blood samples from their cohort prior to resection and 1 month thereafter, and found CTCs present in 66.67% of patients. Further, patients who had a CTC count greater than or equal to two per every 7.5 mL of blood collected developed tumor recurrence earlier than those than those patients who had a CTC count of less than two per 7.5 mL (87.5% versus 15.5% of respective cohorts, *p* < 0.001). Additionally, they found that a preoperative CTC count of greater than or equal to two was an independent prognostic factor for tumor recurrence (*p* < 0.001). Importantly, tumor recurrence in the setting of liver transplantation is critical data in determining a patient’s eligibility for transplantation, or for monitoring postoperation. Bai et al. studied 232 patients with HCC undergoing liver transplantation [71]. They surveyed 149 total proteins extracted from homogeneous tumor cells and stratified based on expression levels and tumor recurrence rates. Amongst them all, they identified calpain small subunit 1 overexpression in the recurrence cohort and that the overexpression was associated with enhanced tumor cell invasiveness in vitro. Notably, siRNA-knockdown expression of Capn4 significantly inhibited the HCC tumor cell invasive ability and multivariate analysis indicated Capn4 as an independent prognostic factor for tumor recurrence in HCC. Chaiteerakij et al. also studied various serum biomarkers in patients who were undergoing liver transplantation [72]. They found that three biomarkers they studied, AFP, lens culinaris agglutinin-reactive AFP, and des-gamma-carboxyprothrombin (DCP), were all significantly associated with HCC recurrence. Notably, this study incorporated the Milan criteria for liver transplantation eligibility to see if there was an improvement in prognostication. They found better risk prediction of HCC recurrence after transplantation when the Milan criteria was added to AFP and DCP biomarkers. Iseke et al. used MR imaging features and three ML models to predict posttreatment HCC recurrence in 120 patients with early-stage HCC who were initially eligible for liver transplant and had undergone treatment by resection, thermal ablation, or transplant [73]. They found that the ML model that incorporated imaging features was better than the ML that incorporated clinical features (AUC: 0.76 vs. 0.68, respectively; *p* = 0.03), but there was no significant difference between the clinical model and the combined model with both imaging and clinical data (AUC: 0.76 vs. 0.76, respectively, *p* > 0.05). This suggests the importance of incorporating MRI data in designing criteria for liver transplant eligibility.

AI has also allowed the facilitation of biomarkers in HCC risk prediction postablation. Sato et al. developed six ML models and a Cox proportional hazard model to predict the risk of HCC recurrence post radiofrequency ablation (RFA) in 1778 patients with treatment-naïve HCC [74]. Their gradient boosting decision tree (GBDT) model had the highest performance, with a Harrel’s c-index of 0.67 from external validation, and it was able to discriminate well in stratifying the external validation sample in various risk groups (*p* < 0.001). The variables that contributed the most to the prediction of recurrence were tumor number followed by serum albumin level and DCP. An et al. assessed three ML models (random forest, SVM, and eXtreme Gradient Boosting) and one liner regression model on 1574 patients with early-stage HCC who were treated with microwave ablation at four hospitals to predict recurrence [75]. All of their ML models performed better than linear regression (*p* < 0.001 for all), and the eXtreme Gradient Boosting model, with nine variables incorporated that included biomarkers, achieved the best discrimination with an AUC of 0.75 (95% CI: 0.72–0.78) in the training set, 0.74 (95% CI: 0.69–0.80) in the internal validation set, and 0.76 (95% CI: 0.70–0.82) in the external validation set. Finally, Ding et al. developed a hybrid ML model to suggest the optimal first treatment approach for patients with single, untreated HCC with 3–5 cm tumors based on the probability of early recurrence (occurring within two years after treatment) [76]. The study used five different algorithms to build their model and found that by suggesting optimal treatment, their hybrid model could reduce the probability of early recurrence from 38.2% to 25.6% (*p* < 0.001).

## 9. Future Directions

Significant potential remains for AI and ML to enhance biomarker detection in the screening, diagnosis, and management of liver cancers. Leveraging the computational capacity of ML and deep learning techniques to combine multimodal biomarkers (i.e., imaging data with laboratory values) may facilitate the identification of a biomarker panel to enhance diagnostic and prognostic accuracy. The success of AI in biomarker detection will depend on access to unfiltered clinical, laboratory, imaging, and potentially genomic data. Engineering the medical infrastructure to facilitate secure and ethical access to this data is critical. The extent to which biomarkers identified by AI will need to be clinically validated is an evolving debate. AI models often do not provide a clinical correlation to identified predictor variables, and multivariate models may need to demonstrate robustness in the case of missing data to facilitate clinical impact in resource-limited environments. Nevertheless, the computational ability of AI to detect imaging features as well as evaluate the performance of existing biomarkers is promising and will likely impact the clinical HCC paradigm. In addition, AI technologies, in conjunction with patient characteristics, have the potential to support personalized medicine by leveraging heterogeneous input data for predictive analysis. By integrating diverse patient information, AI algorithms can generate tailored predictions, risk assessments, and treatment recommendations. This, however, requires addressing challenges such as secure and ethical access to comprehensive personalized data. Nonetheless, AI’s computational abilities hold promise for enhancing biomarker detection and utilizing it to impact the clinical HCC paradigm.

## 10. Conclusions

In conclusion, the use of AI in detecting biomarkers and their implementation for liver cancer diagnosis and management is an exciting and rapidly growing field. Recent literature has shown that AI can effectively predict the risk, diagnosis, staging, prognosis, treatment response, and recurrence of liver cancers with acceptable discriminative power and accuracy. However, it is important to acknowledge that further research and validation are needed to ensure the reliability and generalizability of these AI-based models. Further work needs to be done in order to clinically validate AI in the biomarker and liver cancer space with diverse multi-institutional datasets and thought into its ethical and social considerations before becoming fully integrated into the clinical space. Despite these challenges, AI-based biomarker research in liver cancer holds immense potential for improving patient outcomes and transforming care. Continued research and collaboration are essential to ensure its potential is realized in clinical practice.

## Figures and Tables

**Table 1:** Biomarkers detected through AI techniques in RNA-seq.

Author	Year	Data	HCC Biomarkers Detected	AI Technique	Findings
Kaur et al. [22]	2019	Large-scale transcriptomic profiling datasets with 2136 HCC and 1665 nontumorous tissue samples	FCN3, CLEC1B, and PRC1	ML	Identification of HCC samples in training/validation datasets had accuracies between 93–98% with AUC 0.97–1.0
Gui et al. [23]	2015	Microarray data from 43 HCC and 52 nontumorous samples	MT1X, BM1, and CAP2, TACSTD2	ML: maximum-relevance–minimum-redundancy algorithm	Features had high prediction accuracies, up to 0.905
Gupta et al. [24]	2021	RNA-Seq data from 24 healthy liver samples and 32 HCC samples	PARP2–202, SPON2–203, and CYREN-211	ML: random forest, K-nearest neighbor, naïve Bayes, support vector machine, and neural networks	High values for random forest and support vector machine (sensitivity of 0.968 and 0.944, respectively; specificity of 1 and 1, respectively). AUC of random forest was 0.99
Gholizadeh et al. [25]	2023	493 HCC and 446 nontumorous samples from the Gene Expression Omnibus (GEO)	Diagnostic signature CYP2E1, AKR1C3, AFP as well as the four-gene prognostic signature including SOCS2, MAGEA6, RDH16, and RTN3	ML	AUC in training set was 0.952 and in validation set was 0.941
Zhang and Liu [26]	2021	High-throughput omics data from the Cancer Genome Atlas (TCGA) consortium	6 Optimal gene subsets—with common biomarkers overlapping: SKAP1, EPHB1, STC2, CDHR2, FAM134B, MUC6, PHOSPHO1, and OXT	ML: Adaboost, K-nearest neighbor, naïve Bayes, neural network, random forest, and support vector machine	AUC ranged from 0.993 to 1.00 for the 6 classifiers
Zhao et al. [27]	2020	Clinical data of 377 patients, mRNA data of 371 patients, and miRNA data of 373 patients from the TCGA project	hsa-miR-10b-5p, hsa-miR-10b-3p, hsa-miR-224-5p, hsa-miR-183-5p and hsa-miR-182-5p	ML: random forest algorithm	AUCs for the 5 biomarkers ranged from 0.784 to 0.889

**Table 2:** AI in predicting risk of liver cancers.

Authors	Year Published	Population	Data Used	AI Technique	Findings
Liang et al. [36]	2021	47,945 patients from the National Health Insurance Research Database of Taiwan	Electronic health records, imaging, histopathology, molecular biomarkers	Convolutional Neural Network	AUC for predicting 1-year risk of HCC 0.94 (95% CI: 0.937–0.943)
Singal et al. [37]	2013	442 patients with Child A or B cirrhosis at the University of Michigan	Patient demographics, clinical data, and laboratory values	Random Forest	ML algorithm had a c-statistics of 0.64 (95% CI: 0.60–0.69)
Konerman et al. [38]	2015	Patients from the Hepatitis C Antiviral Long-Term Treatment Against Cirrhosis (HALT-C) Trial	Longitudinal clinical, laboratory, and histologic data	Random Forest and Boosting	AUC for predicting fibrosis progression was 0.79 (95% CI: 0.77–0.81) for random forest and 0.79 (95% CI: 0.77–0.82) for boosting. AUC for liver-related clinical progression was 0.86 (95% CI: 0.85–0.87) for random forest and 0.84 (95% CI: 0.82–0.86) for boosting. Longitudinal ML models had negative predictive values of 94% for the two outcomes
Dawuti et al. [39]	2022	49 patients with liver cirrhosis, 55 with HCC, and 50 healthy volunteers	Urine for surface-enhanced Raman spectroscopy	Support Vector Machines	Urine SERS identified HCC with sensitivity of 85.5%, specificity of 84.0%, and accuracy of 84.8%
Ioannou et al. [12]	2020	48,151 patients with hepatitis C virus-related cirrhosis in the national Veterans Health Administration	Longitudinal data from electronic health records	Recurrent Neural Network	Mean AUC of recurrent neural network models was 0.759 (SD = 0.009) with mean Brier score of 0.136 (S = 0.003). In patients who achieved sustained virologic response, mean AUC was 0.806 (SD = 0.025) and mean Brier score was 0.117 (SD = 0.007)

**Table 3:** Median expression level (transcript per million) of hub genes in each BCLC tumor stage, adapted from Xu et al. [45].

Hub Gene	Stage 0	Stage A	Stage B	Stage C	Stage D
TIGD5	1.17	4.30	5.09	5.73	4.57
C8ORF33	7.46	23.03	24.84	26.60	32.74
NUDCD1	1.79	3.84	4.08	4.40	5.08
INTS8	2.87	7.66	8.78	9.13	6.33
ZNF623	1.17	2.57	2.62	3.51	2.20
STIP1	22.93	56.86	65.24	76.67	64.03
HSP90AB1	196.33	505.67	592.33	684.23	759.80
DSCC1	0.26	1.49	1.67	2.16	1.55
POP1	0.47	1.01	1.20	1.26	1.00
ARHGAP39	0.20	0.67	0.89	0.94	0.71
PRKDC	3.13	6.74	7.83	9.09	5.10
YDJC	5.59	11.14	11.46	13.79	13.03
PUSL1	4.53	9.01	11.60	11.53	11.85

**Table 4:** Predicting efficacy and response to treatment through AI-facilitated biomarkers.

Author	Year	Treatment	Biomarker(s)	AI Technique	Findings
Hsu et al. [54]	2022	Lenvatinib	AFP, albumin–bilirubin (ALBI) grade, and circulating angiogenic factors	ML (RF and decision tree-based survival predictive model)	Reduction in AFP ≥ 40% from baseline within 8 weeks posttreatment was associated with a higher objective response rate (ORR). Patients with high, intermediate, and low ORRs were identified. Baseline AFP was the most important factor in determining OS, followed by ALBI grade and FGF21.
Ma et al. [55]	2023	TACE and Lenvatinib	K, low-density lipoprotein, D-dimer, red blood cell, alanine aminotransferase, albumin, monocyte, tumor size, triglyceride, and age	ML (classification and regression tree, adaptive boosting, extreme gradient boosting, RF, and SVM) and Shapley additive explanation	Their predictive models had AUCs between 0.74 to 0.91. SVM and RF algorithms achieved the highest accuracy rate of 86.5% SHAP model showed that patients with lower serum K, older age, larger BMI, and larger tumor size were more likely to be responsive to the combination therapy.
Zhong et al. [56]	2019	TACE	ALBI grade and CTP score	Nomogram and artificial neural network (ANN)	Their ANN found that ABLI grade had greater significance than CTP score in predicting survival.
Morshid et al. [18]	2019	TACE	CT imaging data and clinical data	ML (RF)	Combined BCLC stage with quantitative image features showed a predication accuracy of 74.2%, while the model with BCLC stage alone had a prediction accuracy of 62.9%.
Abajian et al. [19]	2018	TACE	MR imaging data and clinical data	ML (LR and RF)	Both models could predict treatment response with an overall accuracy rate of 78% The strongest predictors of treatment response included an imaging variable (relative tumor signal intensity >27.0) and a clinical variable (presence of cirrhosis).
Peng et al. [57]	2020	TACE	CT imaging data	DL with a residual CNN (ResNet50)	The DL model demonstrated an accuracy of 84.3% and AUCs of 0.97, 0.96, 0.95, and 0.96 for complete response (CR), partial response (PR), stable disease (SD), and progressive disease (PD). Decision curve analysis (DCA) revealed that the ResNet50 model had a high net benefit in the validation cohorts.
